# Variations in Ischemic Heart Disease Research by Country, Income, Development and Burden of Disease: A Scientometric Approach

**DOI:** 10.15171/jcvtr.2015.35

**Published:** 2015-12-03

**Authors:** Maryam Okhovati, Morteza Zare, Azam Bazrafshan

**Affiliations:** ^1^ Physiology Research Center, Institute of Neuropharmacology, Kerman University of Medical Sciences, Kerman, Iran; ^2^ Neuroscience Research Center, Institute of Neuropharmacology, Kerman University of Medical Sciences, Kerman, Iran

**Keywords:** Ischemic Heart Disease, National Income, Mortality, Burden of Disease

## Abstract

***Introduction:*** Ischemic heart diseases (IHDs) are the leading cause of mortality worldwide. However the global burden of IHD has been concentrated on developing countries, where limited research efforts have been made to address these needs. This study aimed to understand the global distribution of IHD research activities by looking at the countries’ burden of disease, income and development data.

***Methods:*** As a scientometric study, Scopus database was searched for research publications indexed under the medical subject heading (MeSH) ‘myocardial ischemia’ including the following terms: coronary artery disease, coronary heart disease, and ischemic heart disease. The number of research publications in Scopus database was recorded for each individual year 2000-2012, and for each country. Data for estimated IHD disability-adjusted life-year’s (DALY’s), gross domestic product (GDP) per capita and human development index were also included for the analysis.

***Results:*** IHD research publications were most likely produced by European and Western pacific countries. High-income countries produced the greatest share of about 81% of the global IHD research. However, no significant association observed between the countries’ GDP and number of research publications worldwide (OR = 0.98, *P* = 0.939). Global IHD research found to be strongly associated with the burden of disease (*P* < 0.0001) and the countries’ HDI values worldwide (OR = 16.8, *P* = 0.016).

***Conclusion:*** Our study suggested that global research on IHD were geographically distributed and highly concentrated among the world’s richest countries. Estimated DALYs and HDI were found as important predictors of IHD research and the key drivers of health research disparities across the world.

## Introduction

With an estimated 17.5 million deaths, cardiovascular diseases (CVDs) are the leading causes of mortality worldwide.^1^ About 75% of the global CVD mortality occurs in low- and middle-income countries (LMICs). The rates of CVD mortality have been reduced dramatically in some parts of the world, but it still threatens millions of lives in developing countries. While the global burden of CVD has been concentrated disproportionately among the transitional and developing countries, research produced by these countries has been found limited.^2,3^

There is evidence indicating that the volume of research in developing countries on non-communicable diseases (NCDs) has been very poor. More than ninety percent (90.5%) of articles on CVDs research published between 1995 and 2002 in PubMed database were from Western Europe, the United States, Japan, and Canada.^4^ Mendis et al observed that over three quarters of CVD publications in 1991, 1996, and 2001 produced by developed market economies.^2^ Al Kindi et al put forward that despite the Middle East has a high prevalence of non-communicable chronic diseases, a low percent of publications is produced by this area (3%). Of course, the overall trend showed an increase in the number of the articles on CVDs over the years but their publications lag behinds developed countries.^5^ Although according to Bosu about three-quarters of global deaths from NCDs occur in developing countries, less than 10% of research on the CVD comes from these countries.^6^

While it is important to map priorities in medical research to bring them more in line with current needs, several studies investigated whether research publications are in line with the burden of specific diseases^3,7-9^ and preliminary investigations provide further evidence that much regional disparities have been emerged in global health research and many common conditions and diseases have not been studied adequately. According to the Global Forum for Health Research estimation, less than 10% of the global resources have been directed toward health research in developing countries accounting for over 90% of all preventable deaths worldwide.^10,11^

In an attempt to address these challenges, the present study aimed to provide detailed information on the global distribution of research publications particularly on IHD during 2000-2012 in Scopus database. Further analysis has been made to test whether IHD research publications were associated with the global burden of disease, income and development of countries.

## Materials and Methods

This is a scientometric study. In January 2015, Scopus database was searched for research publications indexed under the medical subject heading (MeSH) ‘myocardial ischemia’ including the following terms: coronary artery disease, coronary heart disease, and Ischemic heart disease. The number of research publications in Scopus database was recorded for each individual year 2000-2012, and for each country the aﬃliation of the authors was used for identifying affiliation of a research publication to a country. In this regard, the scientific output of 172 countries extracted from the Scopus database. The ﬁrst year for the search was 2000, because of retrieving reliable data on global burden of diseases data available through World Health Organization (WHO) advisory database.

Data for the global burden of IHD were extracted from the WHO disability-adjusted life-year (DALY) estimates for the year 2012. Data for gross domestic production (GDP) per capita for each country was extracted from the World Bank database. Human development index (HDI) data were also extracted from the United Nations Development Program (UNDP) database.

Using STATA software (version 11.0), multilevel regression analysis (MLRA) was performed to explore the associations between the number of research publications and disease burden by region. Then GDP per capita and HDI were entered into the model to estimate the adjusted association between the number of scholarly articles and disease prevalence. The significance level was 5% for two sided tests.

## Results

Totally, 172 countries were included in the study. Global research on IHD were geographically distributed and highly concentrated among the world’s richest countries (Table 1). About 88% of the research was produced by scientists in 20 countries. The United States was the most productive, contributing to 27% of the global IHD research production. Western pacific region (WPR) indicated the most average contribution to IHD research worldwide (4495.1). However, African countries indicated the least average contribution by 33.9.

**Table 1:** Top 20 IHD Related Research Producers

**Country**	**Total Global Share 2000-2012 (%)**
USA	27.18
China	8.63
United Kingdom	7.08
Germany	6.71
Japan	5.90
Italy	4.87
Canada	3.48
France	3.48
Spain	2.69
Netherlands	2.68
Australia	1.99
Turkey	1.91
Republic of Korea	1.65
Switzerland	1.56
Poland	1.52
Sweden	1.51
India	1.48
Brazil	1.23
Belgium	1.09
Greece	1.04

**Figure 1:**
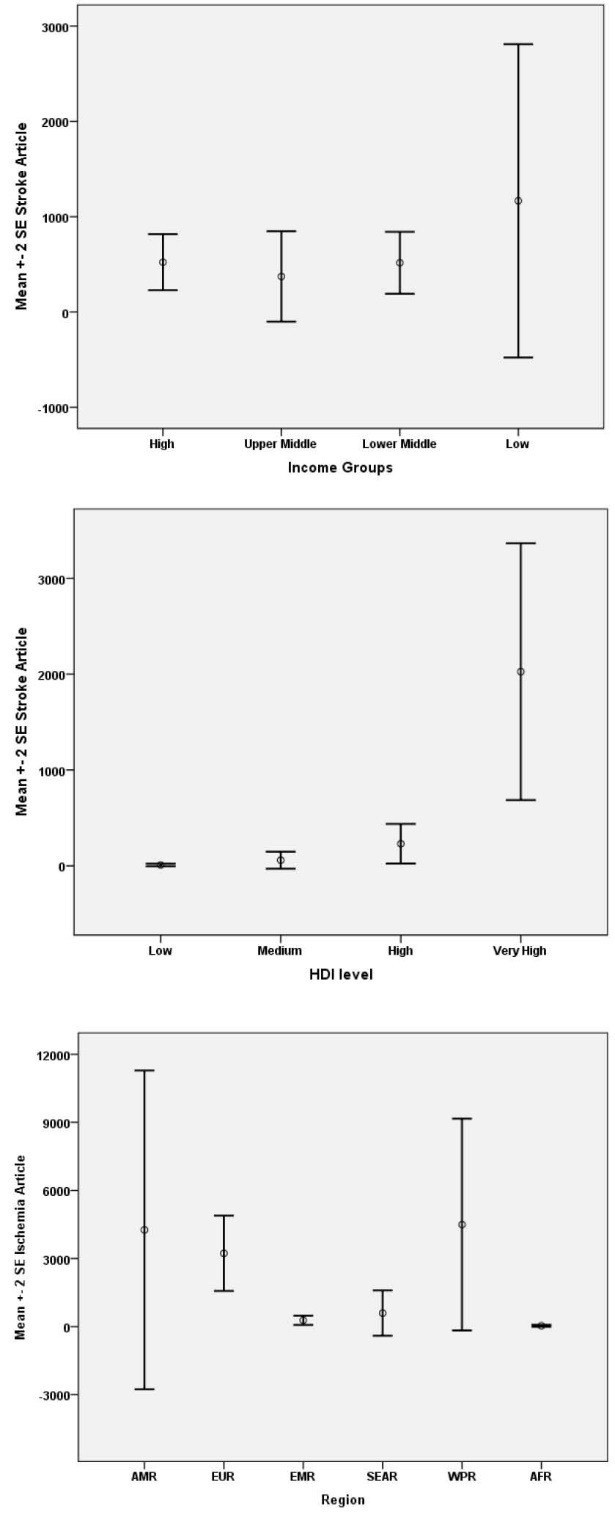
Distribution of Global Ischemic Heart Diseases (IHD) Research across Regional, Income and Development Groups.

Global distribution of IHD research publications across different geographic regions, income and development groups are is illustrated in Figure 1.

From our analysis of global IHD research data, our findings indicated significant variations among geographic regions (*P *< 0.0001; Figure 1). In essence, IHD research publications were most likely produced by European and western pacific countries accordingly (Table 2).

**Table 2:** Multivariate Analysis of IHD Research Publications According to Socioeconomic Determinants of Health

**Variable**	**OR**	*** P *** ** value**	**95% CI**
HDI	16.80	0.016	1.703-165.690
Estimated DALYs	1.0005	<0.0001	1.002-1.008
GDP per Capita	0.98	0.939	0.721-1.351
Region			
Europe	1	‏--	‏--
America	0.15	<0.0001	0.056-0.446
Eastern Mediterranean	0.08	<0.0001	0.032-0.247
South East Asia	0.02	<0.0001	0.005-0.081
Western Pacific	0.62	0.443	0.192-2.026
Africa	0.004	<0.0001	0.001-0.010

High-income countries produced the greatest share of about 81% of the global IHD research during 2000-2012, followed by lower-middle-income (LMI) countries with 12% and upper-middle-income (UMI) countries with 0.07%. In contrast, scientists from LMI countries had the least contribution to the global IHD research. The contribution to the global IHD research differs by country-income groups, as defined by the World Bank in 2013. However, no significant association observed between the countries’ GDP and number of research publications worldwide (OR = 0.98, *P* = 0.939; Table 2).

We found a significant positive association between the countries’ HDI values and number of IHD research publications worldwide (OR = 16.8, *P* = 0.016). Besides, the number of IHD research publications found to be strongly associated with estimated DALYs (*P* < 0.0001; Table 2).

## Discussion

This study estimated the global distribution of IHD research between 2000 and 2012. Data from 172 countries were extracted and the pooled results yielded a broad estimate of the IHD research productivity so far. This approach also allowed us to explore whether the research performance in the country level is more or less associated with the global burden of disease and socioeconomic determinants of health across regions.

Our findings indicated that the United States had the largest contribution in the global IHD research production following by China and the United Kingdom. This finding confirms early evidence that global research on IHD was highly concentrated among the world’s richest countries.^4,12^ It follows the 20/80 law so that about eighty percent of the research was produced by scientists in 20 countries.

WPR indicated the most average contribution to IHD research worldwide, while African countries indicated the least average contribution.

In addition, high-income countries found to have the greatest contribution, followed by LMICs and UMICs. In contrast, scientists from LMICs countries had the least contribution to the global IHD research. However, our findings suggested no significant association was observed between the countries’ GDP and number of research publications worldwide. But a significant positive association between the countries’ HDI values and number of IHD research publications worldwide. Furthermore, the number of IHD research publications found to be strongly associated with estimated DALYs.

Early investigations highlighted the existing disparities between developed and developing countries in terms of CVD research publications.^2,5,12^ The volume of research in developing countries on NCDs has been found very low, reflecting a huge research-funding gap between developed and developing countries. Of 86 711 articles on CVD research published between 1995 and 2002 globally and available from the PubMed database, 90.5% were from Western Europe, the United States, Japan, and Canada. Latin America and the Caribbean accounted for 1.1% of the articles, while Africa accounted for a lower percent (0.3%). It is reported elsewhere that 78%–79% of CVD publications in 1991, 1996, and 2001 came from developed market economies, 6%–8% from developing countries, and 4%–8% from developing Eastern European countries.^2^

In this context, the limited share of health research from national health funds influenced the quantity and even quality of research in developing countries. Previous studies indicated that observational studies formed the major publication group in these countries and the number of publications from clinical trials was extremely limited. The largest proportion of publications related to health system, quality of care, and cost/cost-effectiveness was contributed by high-income countries and the contribution of developing countries in this category was estimated as 3%.^12^

Although recent evidence indicated a dramatic growth (about 130%) in the number of publications related to CVD during the last decades; no significant variation was observed in the share of publications between the high-income and low-income (LMICs and LICs) groups.^12^

According to our findings, the number of published articles across the world was substantially associated with the estimated DALYs. This finding is interestingly highlighting the fact that research on CVDs has been globally directed on the population health priorities. Besides, our findings indicated an important association between the number of research publications and HDI values. Our findings showed HDI to be an important predictor of health research achievements in the IHD area in particular and a key driver of development in the world. HDI is a composite measure of life expectancy, education and Income that emphasizes on the role of people and their capabilities in assessing the development of countries as well as economic growth.^13^ This measure has been developed by United Nations to evaluate and rank countries’ level of social and economic achievements. The HDI tracks changes in development levels over the years and to compare development levels in different countries.

In summary, our study suggested that global research on IHD was geographically distributed and highly concentrated among the world’s richest countries. Estimated DALYs and HDI were the found to be strongly related with the number of research publications across the world.

## Ethical issues

This work has been granted by Ethics Committee of Kerman University of Medical Sciences.

## Competing Interests

The authors declared no conflict of interest regarding this work.
